# Depression and anxiety in family caregivers of patients with schizophrenia in tunisia

**DOI:** 10.1192/j.eurpsy.2023.1756

**Published:** 2023-07-19

**Authors:** D. Njah, M. Lagha, S. Boudriga, W. Homri, I. Ben romdhane, R. Labbene

**Affiliations:** psychiatry “C”, Razi hospital, tunis, Tunisia

## Abstract

**Introduction:**

Around 1% of the general population have schizophrenia. It dramatically affects not only the patients who suffer from it, but also their family members. It represents a difficult task for family caregivers, especially at the time of deinstitutionalization of the patients, when they have to assume some of the functions and care previously provided by psychiatric institutions. This day-to-day care can influence the lives of the caregivers and cause anxiety or depression, which might affect the care that the patients receive.

**Objectives:**

The objectives of our study were to assess anxiety and depression in family caregivers of patients with schizophrenia and to identify associated risk factors.

**Methods:**

We conducted a cross-sectional study including family caregivers of patients with schizophrenia. Anxiety and depression were assessed using the 14-item Anxiety and Depression Scale in its validated version in Tunisian dialect (HAD scale).Statistical analysis was performed by SPSS 26.0.

**Results:**

We included 30 family caregivers of patients with schizophrenia.

The prevalence of depression in family caregivers was 40 % while 56% of them were anxious. Six caregivers had both depression and anxiety, 63.3% of them were unemployed and 52.2% stopped working to take care of their relative.

In our study, the schizophrenic patient’s history of aggression towards the caregiver was statistically associated with depression (p=0.025 ). The worse the compliance of the patient to the treatment, the more likely the caregiver is to develop anxiety (p= 0.027).The parents (mother or father) were the most exposed to depression, anxiety or both (p=0.016). Family caregivers who lived with the patient under the same roof developed more anxious symptoms than the ones who didn’t (p=0.005). The time spent taking care of the patient was higher for the caregivers with depression, anxiety or both (p = 0.046).

**Conclusions:**

Schizophrenia may cause a significant psychological distress for family members such as depression or anxiety .Several factors seem to be involved, inherent to the disease, to the patient and to the caregiver.

**Disclosure of Interest:**

None Declared

